# Plasticity of the Dorsal “Spatial” Stream in Visually Deprived Individuals

**DOI:** 10.1155/2012/687659

**Published:** 2012-08-26

**Authors:** Giulia Dormal, Franco Lepore, Olivier Collignon

**Affiliations:** ^1^Centre de Recherche en Neuropsychologie et Cognition (CERNEC), Université de Montréal, Montreal, QC, Canada H3C 3J7; ^2^Institute of Psychology and Institute of Neurosciences, Université Catholique de Louvain, 1348 Louvain-la-Neuve, Belgium; ^3^Centre de Recherche du CHU Sainte-Justine, Université de Montréal, Montreal, QC, Canada H3T 1C5; ^4^Center for Mind/Brain Sciences, University of Trento, 38060 Mattarello, Italy

## Abstract

Studies on visually deprived individuals provide one of the most striking demonstrations that the brain is highly plastic and is able to rewire as a function of the sensory input it receives from the environment. In the current paper, we focus on spatial abilities that are typically related to the dorsal visual pathway (i.e., spatial/motion processing). Bringing together evidence from cataract-reversal individuals, early- and late-blind individuals and sight-recovery cases of long-standing blindness, we suggest that the dorsal “spatial” pathway is mostly plastic early in life and is then more resistant to subsequent experience once it is set, highlighting some limits of neuroplasticity.

## 1. Introduction

Increasing evidence of experience-based plasticity have challenged the archaic view of the brain as being hard-wired at birth. One of the most striking examples comes from studies on sensory-deprived individuals, documenting that brain regions deprived of their “natural” sensory inputs (i.e., V1 for vision or A1 for audition) in the blind and the deaf brain become responsive to the remaining modalities, a phenomenon that is referred to as *crossmodal plasticity* [1]. Importantly, such plasticity is not restricted to an early sensitive period in life, but rather appears to extent into adulthood. In the case of blindness for instance, individuals who lose vision after the full development of the visual system also present auditory and tactile responses in the deprived occipital regions [2–6].

In the sighted brain, the existence of separate hierarchical pathways for object identification (the ventral “what” stream) and object localization/grasping in space (the dorsal “where” stream) appears as a general principle of organization of the visual cortices [7, 8]. Crucially, recent studies on crossmodal reorganization in the blind suggest that the crossmodal recruitment of the visually deprived cortices in this population might follow organizational principles that maintain this dual stream segregation ([9], for reviews see [10–12]).

In the present review, we focus on the “dorsal stream” and bring together existing evidence suggesting that this stream is, on the one hand, highly plastic in early life and, on the other hand, very resistant to subsequent experience once it is set. Evidence comes from studies on three different visually deprived populations, that is, cataract-reversal patients, early- and late-blind individuals, and rare cases of sight-recovery individuals after long-standing blindness.

## 2. Cataract-Reversal Patients

**Figure 1:**
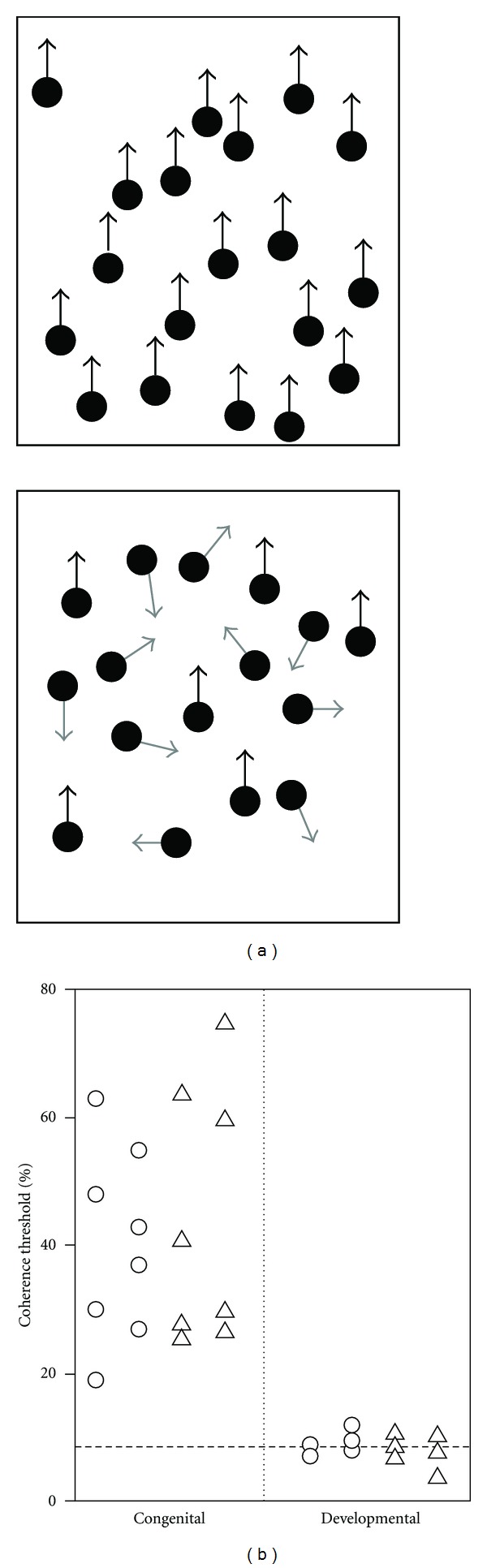
(a) Schematic representation of stimuli used to test the coherence threshold, a typical measure of sensitivity to global motion. Among randomly moving dots, the coherence threshold is the minimal percentage of dots moving in the same direction needed for the participant to accurately perceive this predominant direction of motion. Upper-panel represents a trial with 100% coherence as all the dots are moving in the upward direction. Bottom-panel represents a trial with 37% coherence, as 6 out of 16 dots are moving upward whereas the remaining 10 dots are moving in random directions. (b) Global motion coherence thresholds for each subject in the bilateral congenital and bilateral developmental groups tested in the study of Ellemberg et al. [17]. Circles represent the data from the better eyes and triangles represent the data from the worse eyes. The dashed line represents the mean of 24 sighted control subjects. Adapted with permission from [13, 17].

Studies of individuals who were visually deprived early during development, because they were born or developed dense bilateral cataracts which were then surgically removed, represent a unique model to test the role of early visual experience in shaping the functional architecture of the brain. Such studies have documented the existence of different sensitive periods during which visual inputs are necessary for the normal development of different aspects of vision [13]. Specifically, global motion perception, a visual function associated to bilateral occipitoparietal regions in the dorsal visual stream [14–16], and which allows to integrate local motion information from V1 into a global representation of motion, appears to be permanently altered in cases where vision is absent at birth [17]. However, this function is preserved in cases where the loss of sight occurs after a few months of age [17]. For instance, Ellemberg and colleagues tested global motion perception abilities in a group of bilateral cataract-reversal patients that had been visually deprived at birth and for a period lasting from 3 to 8 months (i.e., congenital group) or in a separate group that had been visually deprived between 8 and 57 months of age, after a period of normal visual experience (i.e., developmental group). The congenital group was found to be strongly impaired compared to the developmental group (Figure 1(b)). In the latter group, all participants performed within normal limits, even in cases where vision had been lost as young as 8 months of age and for a period lasting up to 6 months.

These observations suggest that visual deprivation during the first months of life is sufficient to permanently alter global motion perception abilities, whereas visual experience before the age of approximately 8 months may be necessary and sufficient for the normal development of sensitivity to global motion in humans. Consistent with these findings, kittens raised in the dark up to 4 to 10 weeks of age, showed profound and long-lasting deficits in global motion perception following the period of deprivation, provided it started before 6 weeks of age [18]. Interestingly, the same animals did not show apparent deficits in the perception of simple unidirectional motion, arguing for a larger impact of early visual deprivation on extrastriate than on early visual cortices ([18, 19], but see [20, 21]).

Importantly, studies on the normal development of global motion processing, which is usually considered as an indicator of the dorsal stream maturation [22] suggest that sensitivity to global motion emerges between 6 to 11 weeks of age [23, 24] but reaches adult-like level of performance later in life, although the age of mature performance remains unclear. Some have reported that global motion perception is mature before 3 years of age [25], others between 6 and 11 years of age [17, 26, 27]. A recent study pushes the age of maturity for sensitivity to global motion even later in life, reporting that a group of adults performed equally well than a group of children aged 12 to 14 years old, but significantly better than groups of children aged 6 to 8 years old and 9 to 11 years old [28]. Such discrepancies have been attributed to the different stimuli parameters used in the different studies, such as the dot speed and the dot density [26, 29].

Combining results in cataract-reversal individuals and normally developed individuals seem to indicate that global motion perception can reach adult-like levels of maturity, despite a period of deprivation occurring when the function is not fully developed yet, provided the individual has experienced normal visual input during the early sensitive period starting sometime around birth and lasting within the first year of age [17, 30]. As such, global motion perception is a compelling example of what Maurer and colleagues refer to as a “sleeper effect,” where early visual experience sets up the neural architecture for later normal development [31]. Interestingly, whereas sleeper effects have been documented in this population for other aspects of low- and high-level vision such as grating acuity, contrast sensitivity, and holistic face processing, some aspects of vision commonly associated to the ventral visual pathway do not necessitate early visual input in order to develop normally. For example, specific aspects of face perception such as face discrimination based on the overall contour of the face, face discrimination based on the shape of internal features, facial expression discrimination, eye gaze, and lip reading perception are all abilities that appear to be preserved even in the absence of early visual input [32–35].

 Overall, studies on cataract-reversal individuals constitute a first type of evidence suggesting that sensitivity to global motion, a function related to the dorsal visual pathway, sets up very early in life and is then resistant to subsequent experience.

## 3. Visually Deprived Individuals

Longstanding blindness is another fascinating model to investigate the role of visual experience on brain development. To date, a wealth of studies have documented that visual deprivation leads the visually deprived occipital regions to massively respond to auditory and tactile inputs (for reviews, see [1, 36]). Comparing the profile of blind individuals who were born as such to those who lost sight later in life after several years of functional vision, further allows questioning the role of *early* visual experience and the role of *the total duration of visual experience* in building the functional architecture of specific brain structures. Beyond the general dual-stream organization of the occipital cortex in sighted individuals, a further segregation concerns its subdivision into several functional areas or “modules,” each of which is specialized for a particular aspect of vision. Within these modules, extrastriate dorsal regions, such as hMT+/V5 and hV3d/V3A, have been extensively described as underlying motion perception in the visual modality [14–16]. Interestingly, in blind individuals who have lost vision at birth or soon after birth, the putative homolog of these regions show responses to motion albeit in the auditory [37–40] and in the tactile [41–43] modalities (Table 1). Moreover, activation in response to auditory motion in putative homolog of area hMT+/V5 bilaterally in blind individuals reflects the direction of perceived moving sounds [40], a property that is known to characterize these regions in the sighted brain for visually moving stimuli [44]. Such results further account for the fact that crossmodal activations in response to auditory dynamic stimulation in these regions subserve a functional role in nonvisual motion processing rather than representing unspecific activation [40]. Therefore, these studies have accounted for the idea that crossmodal plasticity associated to sensory deprivation is functionally specific, in the sense that the mapping of auditory and tactile functions onto visually deprived cortices in the early blind brain appears to follow the natural organization of such regions in the sighted brain [10, 11].

In the same vein, several studies using different paradigms and neuroimaging techniques have consistently demonstrated that spatial hearing in the early blind leads to dorsal occipital recruitment, mainly in the right, spatially dominant, hemisphere (Table 1). In a pioneer PET study, Weeks and colleagues showed that sound localization strongly activated association areas in the right dorsal occipital cortex of early blind individuals but not sighted controls [47]. Another PET study extended these findings, identifying a network of regions in the right dorsal extrastriate cortex that was activated when early blind individuals, but not sighted controls, performed a monaural sound localization task [45]. Further, the functional relevance of such recruitment was ascertained by the fact that several foci of this network correlated with sound localization performance: the blind individuals with the highest performance were the ones who activated these regions the most [45]. Another study suggested that specific recruitment of right dorsal occipital regions in early blind individuals might be present for spatial processing not only of auditory but also of tactile inputs [46].

**Figure 3:**
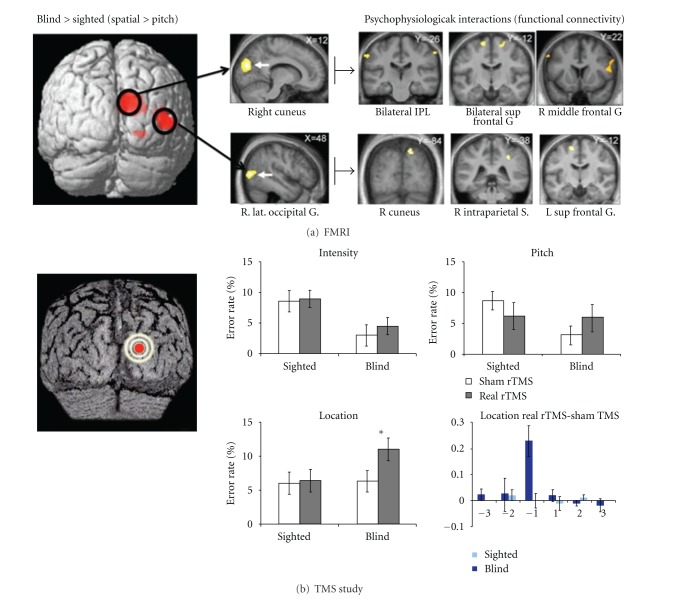
(a) The left part of the figure illustrates the activations obtained in the study of Collignon et al. [38] from the contrast testing which regions are specifically dedicated to the spatial processing of sounds in early blind subjects relative to sighted controls: [Blind > Sighted] × [Spatial > Pitch]. Functional data are overlaid (uncorrected *P* < 0.001) over a 3D render of the brain (left is left). The right part of the figure displays psychophysiological interaction results using the two main activity peaks as seed areas. (b)The 3D brain representation (left is left) displays the projection of the site of TMS application in the study of Collignon et al. [51]. This area corresponds to the right dorsal extrastriate occipital cortex (BA 18). The histograms denote the average error rate in early blind and sighted subjects after sham (control) and real rTMS targeting the dorsal occipital stream during auditory tasks involving the discrimination of intensity, pitch and spatial location of sounds. The data show a significant increase of the error rate after real rTMS only in the early blind group and selectively for the sound location task. The histogram on the right bottom of the figure represents the percentage of errors in the spatial location task in early blind and sighted subjects for the real rTMS condition minus the sham TMS condition (isolating the effect of the TMS), as a function of sound position. Negative values on the *x*-axis are referring to the left external space, positive values on the *x*-axis are referring to the right external space. Adapted with permission from [38, 51].

 In a recent fMRI study, we characterized brain activity in early blind and sighted individuals while they were performing discrimination tasks on pairs of sounds differing either in terms of location in space or in pitch [38]. In this study, a staircase paradigm was used in order to equalize difficulty levels across tasks and participants. The spatial localization task relative to the pitch discrimination task was shown to preferentially map onto specialized subregions of the right dorsal occipital stream in the early blind group but not in the sighted group (Figure 3(a)). The two mainly recruited regions were the right cuneus and the right middle occipital gyrus, in the vicinity of regions corresponding to the dorsal hV3d/V3A and hMT+/V5 in the sighted (Figure 3(a)). Interestingly, these two regions have been extensively described as subserving visuo-spatial and motion processing in the sighted brain [14–16, 48]. Although the task involved auditory localization rather than motion processing, we hypothesize that hMT+/V5 was activated because the task generated a vivid perception of apparent motion [38]. Functional connectivity analyses demonstrated that these occipital regions are part of a larger parietofrontal network including multisensory regions (i.e., the inferior parietal lobules, the intraparietal sulcus and the superior frontal gyrus) that are typically involved in spatial attention and awareness in the sighted brain [49] (Figure 3(a)). Hence, in the absence of visual experience since birth, crossmodal reorganization might be constrained to regions characterized by the same functional specificity, accounting for the fact that these dorsal occipital regions are strongly connected to an extended brain network wired to serve a specific function.

Transcranial magnetic stimulation (TMS) studies further account for the functional relevance of the right dorsal occipital recruitment observed for spatial hearing in the early blind [50, 51]. In an offline TMS paradigm, stimulation applied over these regions led to subsequent alteration of performance in an auditory spatial localization task in early blind but not in sighted individuals, whereas performance in the pitch and intensity discrimination tasks remained unaffected in either group [51] (Figure 3(b)). Most interestingly, the detrimental effect of TMS in the early blind group during the spatial localization task was massively driven by a disruption in the ability of blind individuals to locate sounds presented at the closest position relative to the reference sound in the contralateral (i.e., left) field relative to the right-sided site of stimulation (Figure 3(b), right bottom histogram). This is highly consistent with evidence from the sighted literature documenting a contralateral field preference in several visual areas along the dorsal pathway including hV3d/V3A and hMT+/V5 [15, 48, 52–54]. These results further stress the idea that crossmodal recruitment of the dorsal stream in early blind individuals is functionally relevant and somehow follows the same computational constraints as those observed in sighted individuals when they process visual inputs.

**Figure 2:**
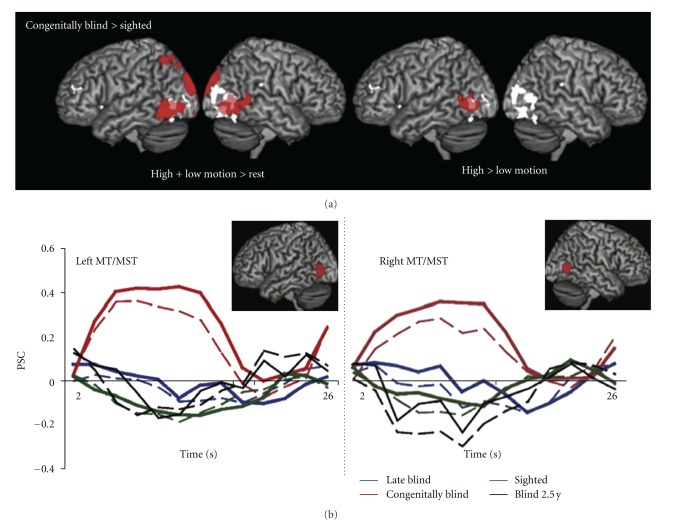
(a) Red colored regions denote activations obtained in the study of Bedny et al. [37] from the contrast testing which regions show greater BOLD signal in response to auditory motion in the congenitally blind relative to the sighted controls, in high and low motion conditions relative to rest (left panel) and in the high motion condition relative to the low motion condition (right panel) (*P* < 0.05, corrected). White colored regions are activated in a sighted group of controls when viewing moving relative to stationary dots. The overlap between regions activated during auditory motion perception in the congenitally blind relative to the sighted, and the ones activated during visual motion perception in the sighted are colored in pink. (b) Percent signal change relative to baseline as a function of time (seconds) in response to auditory motion is displayed in left and right MT/MST ROIs (overlaid in red on a normalized template (left is left) and identified by means of a visual motion localizer in sighted participants) for sighted, congenitally blind, and late blind participants. Solid lines represent percent signal change in response to the high motion condition (footsteps) and dashed lines represent percent signal change in response to the low motion condition (tones). Adapted with permission from [37].

The existence of a critical period in order for sound processing to lead to specific crossmodal recruitment of the dorsal visual pathway was recently suggested. For instance, Bedny and collaborators defined bilateral hMT+/V5 by means of a classical visual localizer in a group of sighted individuals (Figure 2(a), white) and demonstrated that these regions responded to moving sounds in a group of congenitally blind individuals but not in the sighted control group (Figures 2(a) and 2(b), red color), neither in a group of five late blind individuals who lost sight after 9 years of age (range: 9 to 34), (Figure 2(b), blue color) [37]. Interestingly, these regions did not present any response to moving sounds in an early-blind individual who had functional vision until he lost it between 2 and 3 years of age (Figure 2(b), black color). In this latter individual, activation evoked by moving sounds in hMT+/V5 was not higher than in any participant in the late blind group and was lower than in each of the participants in the congenitally blind group. Importantly, the total years of blindness in the late blind participants did not predict the amount of response to auditory motion in this region. It is, however, important to note that in this study, auditory motion stimuli, consisting of footsteps (i.e., high motion condition) or tones (i.e., low motion condition) differing in many low-level properties, were either compared to one another, or compared to a rest condition (i.e., scanner noise). Hence, the specificity of hMT+/V5 activity to auditory motion “*per se*” (rather than to the complexity of the sounds) cannot be ascertained. We recently tested late blind individuals who lost sight after 7 to 51 years of functional vision using the staircase paradigm described above [55]. Whereas massive crossmodal recruitment of occipital cortex to auditory processing, irrespective of the task at hand, was found in late blind individuals, the regions in the right dorsal stream that were preferentially activated for the spatial processing of sounds in congenitally blind individuals did not show any functional preference in blind individuals who lost sight later in life [55].

Taken as a whole, these observations are suggestive of an early sensitive period during which the absence of visual input drives dorsal regions to develop specific crossmodal responses to spatial/motion cues, whereas normal visual input prevents the development of such crossmodal responses, despite years of blindness.

## 4. Sight-Recovery Individuals

Similar conclusions can be raised from rare cases of sight-recovery individuals after longstanding blindness. Two of such cases, SB and MM, have been quite extensively described in the scientific literature. SB lost effective sight at 10 months of age and received a corneal graft after fifty years as a blind person [56, 57]. MM was blind since the age of 3 years old and received stem-cell transplant in his right eye at the age of 46 [58–60]. SB and MM presented striking similarities in their visual abilities following sight restoration. Despite the fact that their retinas regained some functionality, they both encountered extreme difficulties interpreting what they saw, suggesting these deficiencies were from central rather than from peripheral origins. Although they could recognize colors and simple shapes quite accurately, recognition of complex shapes, including faces and everyday life objects, perception of depth cues as well as detection of illusory contours were all abilities that were highly altered. In MM, these visual deficits were further accounted by neuroimaging evidence showing a massive reduction of activation to faces and objects in the fusiform and lingual gyri bilaterally (i.e., the brain areas usually devoted to object and face perception) [58]. Seven years after the intervention, he still had poor spatial resolution and limited visual abilities that prevented him from efficiently relying on his vision in every day life [59, 60].

In contrast to these marked difficulties encountered by SB and MM, motion perception abilities appeared to be quite well preserved in both cases despite years of blindness. MM for instance, performed within normal limits in several motion tasks, whether he had to detect the direction of a moving pattern, or perceive the orientation or the shape of a moving object. Similarly, Gregory and Wallace reported that SB was only able to recognize certain objects in the environment provided they were moving [56, 57]. As such, motion cues constituted information on which these patients could rely more confidently in order to use their newly acquired vision in their day-to-day activities. Consistently with these preserved motion perception abilities, fMRI measures in MM documented normal size of area hMT+/V5 and normal activation in response to moving versus stationary visual stimuli when tested within months following sight restoration [58] as well as 7 years later [60].

Hence, in marked contrast to deficiencies observed in several aspects of vision, the preservation of motion perception abilities in both patients accounts for the idea that such abilities might have developed with early visual experience. Moreover, it appears that such abilities do not require prolonged visual experience in order to crystallize, as opposed to more ventral-related visual functions such as face and object perception [13]. However, an alternative account is that such cases possessed residual visual capacities for motion perception during the extended period of visual deprivation. Indeed, the vast majority of operable blind individuals are cases of “near-blindness” in the sense that, for blindness to be operable, the retina and eye tissues must be at least partially functional [56, 57]. In cases where blindness is strictly total, that is, where both eyes are insensitive to light, the successful outcome of surgery is minimal. Hence, rare cases of sight-recovery individuals are cases that present at least light perception and maybe also crude motion perception in at least one eye. For instance, one of the last medical records of SB before he received corneal grafts indicated that the left eye was reduced to light perception and showed a normal pattern of retinal vessels, whereas the right eye was capable of perceiving hand movements [56, 57]. Similarly, Ackroyd and colleagues reported that HD, an early-onset blind woman who partially recovered sight at the age of 27, was still capable of perceiving moving shadows during the period she was blind [61]. No such records of medical history previous to surgery are provided in the published reports regarding MM, neither for other more recent cases of sight-recovery individuals following years of congenital blindness [62, 63]. The preserved motion perception abilities observed following years of blindness in such cases might thus be, at least partially, explained by the fact that some dynamic information was still available to them after the onset of visual deprivation [62].

In other words, even a brief period of vision after birth or the maintenance of crude visual abilities in these visually deprived individuals may be sufficient to appropriately tune the motion system. Therefore, in sight-recovery individuals, motion cues are likely to play an important role in rehabilitation, guiding the individual to improve learning of other, more disrupted, visual abilities such as object and face recognition. This is well illustrated by a study of Ostrovsky and colleagues, who reported the cases of three supposed congenital blind individuals whose vision was partially restored after years of blindness [63]. Following cataract removal, similarly to SB and MM, these individuals presented marked difficulties in form and depth perception when looking at static images. However, the introduction of motion cues immediately improved these perception abilities. Further and most importantly, when these subjects were tested several months following partial vision restoration, they could recognize static images that they previously could not recognize unless motion cues were provided. These observations suggest that motion perception abilities, because they were spared as compared to other visual abilities such as form perception, might have guided visual learning for these latter, altered abilities [63].

## 5. Involvement of the Dorsal Occipital Stream for Nonvisual Spatial Processing in the Sighted

A growing body of evidence suggests that occipital cortices participate in processing information from other nonvisual modalities not only in visually deprived individuals but also, to some extent, in the normal sighted brain. For instance, modulatory effects of auditory or tactile motion on visual hMT+/V5 responses have been previously documented in typically developing individuals as a result of multisensory integration [64, 65]. Beyond multisensory processes, it appears that visual cortices in the sighted brain can also be modulated by nonvisual stimuli presented alone. On the one hand, several studies have documented crossmodal *activation *of extrastriate dorsal visual regions by auditory and tactile motion/spatial information in sighted individuals ([41, 42, 66, 67], for a review see [68]). When similar crossmodal activation of “visual areas” has been observed in both blind and sighted subjects, some have interpreted it as evidence for the metamodal/supramodal nature of the brain [69, 70]. It should be stressed that similar activations might actually subserve completely different mechanisms in these two populations. For example, recruitment of visual areas during nonvisual processing could be mediated by visual imagery through top-down mechanisms in the sighted brain ([68, 71, 72], for a review, see [73]) whereas it might subserve nonvisual processing *per se *in the blind brain.

**Figure 4:**
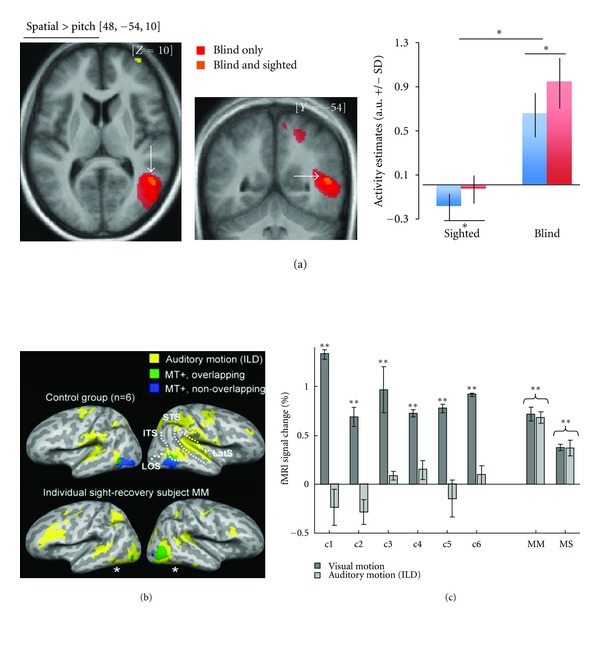
(a) The left part of the figure illustrates the activations obtained in the study of Collignon et al. [38] from the contrast testing which regions are more active for the spatial processing rather than the pitch processing of sounds ([Spatial > Pitch]) in early blind subjects only (red), and in both early blind and sighted participants (orange). Functional data are overlaid (uncorrected *P* < 0.001) over a 3D render of the brain (left is left). The right part of the figure shows beta parameter estimates relative to baseline for the spatial and the pitch conditions, in the sighted subjects and in the early blind participants at coordinate [48, − 54, 10]. (b) Activations obtained in the study of Saenz et al. [60] in six controls subjects (upper part of the figure) and in sight-recovery individual MM (lower part of the figure). Yellow colored regions show a positive difference when contrasting auditory motion to its static condition. Green and blue regions illustrate the overlap and non-overlap with visually defined MT+ in the same subjects. (c) In the same study, percent signal change in visually defined MT+ ROIs is plotted for moving relative to stationary visual stimuli and for moving relative to stationary auditory stimuli in six control sighted subjects (c1–c6) and in two sight-recovery individuals (MM and MS). Adapted with permission from [60].

On the contrary, other studies have shown that nonvisual processing in the sighted brain is associated to *deactivations* in extrastriate visual regions [74, 75]. In fact, within the studies mentioned in the previous sections of this review, some have reported deactivations in the brain of their sighted control participants in area hMT+/V5 during auditory motion processing (Figure 2(b), green color) [37, 60] and in dorsal extrastriate visual areas during auditory and tactile spatial localization [38, 45, 46]. It is worth noting that such crossmodal deactivations observed in the sighted brain might in fact be task dependent [75]. In our staircase paradigm described earlier on, we found significant positive differences in the vicinity of hMT+/V5 when contrasting the spatial and the pitch discrimination condition (i.e., Spatial > Pitch) in sighted participants [38]. However, when plotting the activity estimated in this region, it appeared that both pitch and spatial processing of sounds deactivated (as compared to baseline) this region in sighted subjects (Figure 4(a)). Because the deactivation was found to be greater in the pitch condition relative to the spatial condition, it led to a positive value when contrasting the two conditions (Figure 4(a)). A recent study reported similar observation in a sighted group of participants when plotting activation estimates in their tactile localization and identification conditions in the right middle occipital gyrus [46]. Interestingly, in our study [38], the region showing spatial specificity in terms of deactivation in sighted participants overlapped with the region showing spatial specificity in terms of activation (i.e., functionally specific crossmodal responses) in our early blind group (Figure 4(a)). The voxels with the highest significant difference between the spatial and the pitch conditions in our sighted group was found to be in close vicinity to the coordinates reported in another study when contrasting a condition of moving sounds to a condition of stationary sounds (Moving > Stationary) in sighted participants [76]. Again, a positive difference was reported in the latter study, but because the mean parameter estimates were not displayed separately for each condition in the sighted participants, such positive difference might also putatively result from less deactivation rather than from more activation in the “spatial” auditory task relative to the “stationary” auditory task [76].

Saenz and colleagues reported specific crossmodal responses to motion in two sight-recovery subjects and not in six control sighted subjects based on the observation of significant positive differences between auditory moving and auditory static sounds in these subjects ([60], Figures 4(b) and 4(c)). Evidence of coexisting specific auditory and visual responses to motion relative to their static version in these regions in patient MM who lost sight at 3 years old, is contradictive to results reported by Bedny and colleagues where putative homolog of these regions in a blind who lost efficient sight between the ages of 2 and 3 years old did not develop such crossmodal responses. This apparent contradiction was accounted by the possibility of individual differences in the sensitive period for the development of functionally specific crossmodal plasticity [37]. However, because parameter estimates in these regions were reported systematically for the motion (experimental) conditions relative to their respective static (control) conditions (i.e., motion minus static), it is not possible to disentangle whether this positive difference observed for moving sounds relative to stationary sounds is the result of differences in activations or of deactivations [60].

 Taking these observations as a whole, it was suggested that both activations and deactivations identified during nonvisual tasks can indicate the presence of nonvisual inputs in the occipital cortex of sighted individuals [77]. In agreement with this, TMS studies have demonstrated that disrupting dorsal extrastriate occipital regions in the sighted brain might impair the processing of auditory spatial information [78] and tactile flow [79, 80]. In the sighted brain, existing cortico-cortical connections between auditory and visual cortices [81–84] might play a role for the integration of spatial information coming from different modalities or for the inhibition of visual cortices during nonvisual tasks in order to minimize potential effect of interference with auditory processing [74, 75]. Understanding how specific nonvisual tasks may decrease or increase the activity of the occipital cortex in sighted individuals, and how these crossmodal influences relate to the plastic changes observed in early- and late-blind individuals remains one of the most important challenges for future research in the field.

## 6. Conclusion

Based on the evidence presented here, it could be suggested that early visual experience plays a crucial role for dorsal regions to develop specific visually driven responses to motion/spatial cues, in agreement with the existence of an early and short sensitive period for the normal development of global motion perception in the visual modality. As stressed by studies on cataract-reversal patients, if deprived from normal visual input in the first months after birth, these visual abilities might never develop normally later on [17]. As a direct counterpart, studies on visually deprived individuals have demonstrated that early- but not late-onset blindness drives dorsal regions to develop specific crossmodal responses to spatial/motion cues, maintaining their function for motion/space perception when processing inputs from the remaining modalities [37–43, 45–47]. Finally, the relative preservation of motion perception abilities in sight-recovery patients with functional vision early in life also suggests that such abilities might have developed with early visual experience and do not require prolonged visual experience in order to crystallize [58, 60]. Altogether, these observations are compatible with the idea that the *functional* and *modality* specificity of the dorsal pathway is set early in development and is quite resistant to later acquired experience.

## Figures and Tables

**Table 1:** Summarizing table of brain coordinates (in MNI space) reported in PET and fMRI studies that investigated nonvisual spatial/motion processing in early blind individuals.

Study	Task	Coordinates in MNI space
Striem-Amit et al., [9]	Location and form identification using a visual-to-auditory sensory substitution device (vOICE).	[Location > Form] × [Blind > Sighted]—whole brain
		No activated clusters.
		[Location > Form] in Blind—whole brain
		No activated clusters in occipital and occipitotemporal cortices.

Bedny et al., [37]	Direction of motion judgment performed on receding and approaching moving sounds.	[High > Low motion] × [Blind > Sighted]—whole brain
		L inferior temporal gyrus [−44, −72, −6]
		L middle occipital gyrus [−38, −68, 2]

Bonino et al., [41]	One-back spatial discrimination task performed on 2- and 3-dimensional tactile matrices.	[2D > 3D] in Blind—whole brain (only occipital/occipitotemporal activations are reported)
		L cuneus [−19, −97, −3]
		L middle occipital gyrus [−51, −75, 25]
		L middle occipital gyrus [−35, −90, 11]
		R middle occipital gyrus [38, − 85, 1]
		R middle occipital gyrus [31, −87, 27]
		L lateral occipital [−40, −60, −20]

Collignon et al., [38]	Spatial discrimination versus pitch discrimination of pairs of sounds.	[Spatial > Pitch] × [Blind > Sighted]—whole brain
		R cuneus hV3/V3A [12, −80, 22]
		R middle occipital gyrus hMT+/V5 [48, −76, 6]
		R middle occipitotemporal gyrus hMT+/V5 [40, −56, 12]
		R Lingual gyrus [24, −48, −8]

Gougoux et al., [45]	Binaural and monaural sound localization.	[Monaural localization > Control ] × [Blind with superior performance > Sighted]—whole brain
		R cuneus [13, −81, 15]
		R lingual gyrus [15, −73, −6]
		L cuneus [−13, −79, 9]

Matteau et al., [43]	Motion detection task performed on moving versus static tactile stimuli delivered to the tong using a sensory substitution device, the tong display unit (TDU).	[Motion > Static] in blind—whole brain analyses (only occipital/occipitotemporal activations are reported)
		R middle occipital gyrus [20, −88, 22]
		R middle temporal gyrus hMT+/V5 [42, −54, −4]
		L middle temporal gyrus hMT+/V5 [−44, −64, 2]

Poirier et al., [39]	Motion detection task performed on horizontally moving sounds versus static sounds presented at different locations.	[Motion > Static] × [Blind > Sighted]—ROIs analyses
		R cuneus V3/V3A [24, −88, 10]
		L cuneus V3/V3A [−14, −84, 38]
		R V1/V2 [2, −82, −8]
		L V1/V2 [−24, −88, −8]
		[Motion > Static] in blind (= Sighted)—ROIs analyses
		R Inferior temporal gyrus hMT+/V5 [44, −72, −2]

Renier et al., [46]	One-back spatial versus frequency discrimination task performed on auditory and tactile stimuli.	[Spatial > Frequency] in Blind—whole brain—(only occipital activations are reported)
		R middle occipital gyrus [51, −66, −10]

Ricciardi et al., [42]	Passive tactile perception of moving versus static Braille-like dot patterns.	[Motion > Static tactile] in Blind—whole brain— (only occipital/occipitotemporal activations are reported)
		R hMT+/V5 [38, −69, 7]
		L hMT+/V5 [−45, −82, 5]
		R LOC/LOtv [37, −55, −10]
		L LOC/LOtv [−51, −65, −9]
		V1/V2 [0, −92, −2]
		R V1/V2 [37, −88, −4]
		R cuneus V3A [8, −98, 20]

Weeks et al., [47]	Auditory spatial localization task.	[Localization > Rest] × [Blind > Sighted]—whole brain
		R superior occipital gyrus [22, −79, 22]
		R inferior occipital gyrus [40, −70, −9]

Wolbers et al., [40]	Deviant target detection task within blocks of horizontally moving versus static sounds.	[Moving > Static sounds] in Blind—whole brain (only individual data in occipitotemporal cortices are reported)
		Blind 1
		[−39, −61, 7]; [−54, −64, −2]; [39, −55, 10]; [42, −64, 10]
		Blind 2
		[−39, −67, 4]; [−42, −70, 16]; [45, −64, 10]
		Blind 3
		[−33, −76, 19]; [−48, −76, 13]; [39, −55, 10]; [45, −73, 16]
		Blind 4
		[−48, −73, 13]; [−42, −61, 13]; [42, −64, 1]
